# Maximizing Heterogeneous Server Utilization with Limited Availability Times for Divisible Loads Scheduling on Networked Systems

**DOI:** 10.3390/s23073550

**Published:** 2023-03-28

**Authors:** Xiaoli Wang, Bharadwaj Veeravalli, Xiaobo Song, Kaiqi Zhang

**Affiliations:** 1 School of Computer Science and Technology, Xidian University, Xi’an 710071, China; 2 Department of Electrical and Computer Engineering, National University of Singapore, 4 Engineering Drive 3, Singapore 119077, Singapore; 3 The 20th Research Institute of China Electronics Technology Group Corporation, Xi’an 710068, China

**Keywords:** multi-installment scheduling, server available times, divisible load, networked system

## Abstract

Most of the available divisible-load scheduling models assume that all servers in networked systems are idle before workloads arrive and that they can remain available online during workload computation. In fact, this assumption is not always valid. Different servers on networked systems may have heterogenous available times. If we ignore the availability constraints when dividing and distributing workloads among servers, some servers may not be able to start processing their assigned load fractions or deliver them on time. In view of this, we propose a new multi-installment scheduling model based on server availability time constraints. To solve this problem, we design an efficient heuristic algorithm consisting of a repair strategy and a local search strategy, by which an optimal load partitioning scheme is derived. The repair strategy guarantees time constraints, while the local search strategy achieves optimality. We evaluate the performance via rigorous simulation experiments and our results show that the proposed algorithm is suitable for solving large-scale scheduling problems employing heterogeneous servers with arbitrary available times. The proposed algorithm is shown to be superior to the existing algorithm in terms of achieving a shorter makespan of workloads.

## 1. Introduction

The convergence of information technology and economic society has led to the rapid growth of data. In turn, big data has also changed the traditional production mode and economic operation mechanism [1]. While the big data industry has become a new economic growth point, it has also brought a brand-new challenge: how to effectively and rapidly process large-scale data so as to efficiently mine the value of big data. The development of high-performance networked systems has brought a significant data processing problem [2]. However, how to reasonably allocate workloads on multiple servers directly determines both the resource utilization of networked systems and the processing efficiency of big data. Hence, finding an optimal task-scheduling strategy is the main focus and difficulty of studying networked systems and big data.

The Divisible-Load Theory (DLT) [3] is one of the task-scheduling theories developed under this background. The DLT assumes that a big data workload can be divided into load partitions of arbitrary sizes, without any data dependency or execution order between these load partitions, that is, each load partition can be independently transmitted and processed [4]. These divisions are distributed to multiple servers on the networked system through a reasonable task-scheduling strategy to complete parallel computing, thus shortening the makespan of the entire workload. The DLT has been successfully applied in various big data-related fields, such as image processing [5], dynamic voltage and frequency regulation [6], signature searching [7], data flow optimization [8], real-time video encoding [9], and other typical big data application problems.

It has been proved that divisible-load scheduling problems in networked systems are NP-hard [10]. If the scheduling model is too idealistic, the obtained solution of the model (i.e., load-partitioning scheme) may be difficult to apply to the actual networked systems. On the contrary, if the scheduling model is too detailed with all possible factors that affect the makespan, it will sharply increase the complexity of the problem to be solved, resulting in an inability to obtain an optimal solution to the model within a tolerable time. Therefore, when establishing a task-scheduling model for networked systems, it is necessary to balance the complexity of the model and the performance of the system.

To better adapt to different network topologies of networked systems, various task-scheduling models based on the DLT have been studied. For example, divisible-load scheduling models have been applied on the bus network [11], multi-level tree network [12], Gaussian, mesh, torus network [13], complete b-Ary tree network [14], Cloud platform [15], time-sensitive network [16], wireless sensor networks [17], Edge platform [18], Fog platform [19], and so on.

In order to make task-scheduling models closer to the real distributed platform environment, researchers proposed considerable divisible-load scheduling models with all sorts of constraints. For example, the authors in [20] introduced the concept of startup overheads in the scheduling model, and studied the unneglectable impact of startup overheads on the makespan of workloads. The work in [21] took limited memory buffers into consideration. It assumed that the amount of memory available on the remote servers is too small to hold the whole workload at once. Hence, the workload must be distributed into multi-installments. The authors in [22] studied divisible-load scheduling among multi-task servers whose processing speeds and channel speeds are time-varying. Two recursive algorithms were provided to solve this problem when the arrival and departure times of the background workloads are known a priori and an iterative algorithm was provided to solve the case where such times are not known. The work in [23] focused on heterogeneous networked systems with hierarchical memory. It found that different levels of memory hierarchy have different time and energy efficiencies. Core memory may be too small to hold the whole workload to be processed, while computations using external storage are expensive in terms of time and energy. In order to avoid the costs of processing the workload in the external memory, it allows the workload to be distributed into multi-installments. The authors in [24] studied the communication and computation rate-cheating problems as in a real distributed environment, wherein servers may cheat users by not reporting their true computation or communication rates. The work in [25] addressed failures on servers and takes checkout start-up overhead and checkout time consumption into account. The authors in [26] proposed a novel architecture of a multi-cloud system that can satisfy the complex requirements of users on computing resources and network topology, as well as guarantee a quality of services. Based on this architecture, they designed a dynamic scheduling strategy that integrates the DLT and node ready-time prediction techniques to achieve high performance. The work in [27] studied a scheduling problem with divisible loads and subcontracting options with three linear subcontracting prices. The objective is to minimize the sum of the total weighted tardiness and subcontracting costs. The article in [28] is concerned with the investigation of adapting a user’s preference policies for scheduling real-time divisible loads in a cloud computing environment.

However, the above scheduling models all assume that servers in the system remain idle at the beginning of workload division and assignment and that servers involved in workload computation are able to stay online forever. That is to say, existing studies do not consider the available time of each server. In the actual parallel and networked systems, on the one hand, servers may still be busy computing their previous workload assignment when a new workload arrives. On the other hand, the platform cannot ensure that all servers remain online during workload computation. Servers may become offline or even be shut down before completing their assignment due to network attacks or communication interruptions. In addition, the servers’ continuous operation may cause their temperature to rise due to electric heating, which may endanger the service life of server components; therefore, servers require to be offline periodically to cool down. Meanwhile, servers may also generate available-time fragments due to the user’s advance reservation. If these time fragments are not utilized properly, the overall resource utilization of the servers will be reduced to some extent. On the contrary, if one prefers to make full use of these fragments, then the constraints of server online times and offline times must be considered when allocating load partitions on servers.

From the above analysis, we can see that servers may have heterogeneous available and unavailable time periods for various reasons. Here, available time corresponds to the time period between the server’s release time and the server’s offline time. If we inadvertently assign tasks to servers without considering their availability constraints, all the unfinished workload partitions need to be reassigned to other available servers, resulting in an inefficient time schedule. Hence, limited server available times must be considered when scheduling workloads on a networked system. However, it has been proved that even without server availability constraints, the divisible-load scheduling problem under networked systems is also an NP-hard problem [29].

As regards the server release times alone, several load-scheduling strategies were proposed. For example, the work in [30] addressed the problem of scheduling a computationally intensive divisible load with server release times and finite size buffer capacity constraints in bus networks. The work in [31] addressed divisible-load scheduling with server startup overheads and release times, while a new divisible-load scheduling model was proposed in [32], considering server distribution sequence and release times. The authors in [33] investigated the problem of scheduling multiple divisible loads in networked systems with arbitrary server release times and heterogeneous processing requirements of different workloads. In order to obtain a global optimal load scheduling strategy on tree networks, an exhaustive search algorithm was proposed in [34], but as expected, exhaustive algorithms are time-consuming, especially when networks become large. To make it more time-efficient, the work in [35] proposed a genetic algorithm. In addition to the research based on server release times, the authors in [36] studied the impact of both server online times and offline times, that is, server available times, on the process of divisible-load scheduling.

However, the above studies are all based on single-installment scheduling, which has been proven to be not as efficient as multi-installment scheduling in minimizing the makespan of workloads [10]. In single-installment scheduling models, the master divides large-scale workloads into load partitions with the same number as the slave servers and it only assigns load partitions to each slave server once. By contrast, in multi-installment scheduling models, the master divides workloads into load partitions that are several times the number of slave servers and assigns load partitions to each server in multiple rounds. It is thus clear that, compared with single-installment scheduling, multi-installment scheduling can reduce the waiting time of servers, so it can achieve a shorter makespan and higher utilization of platform resources.

In summary, on the one hand, it is known that multi-installment scheduling is superior to single-installment scheduling in terms of time efficiency; on the other hand, limited server available times should be considered when scheduling workloads on a networked system, but in the existing research, available time constraints have only been studied in single-installment scheduling models. Based on this background, in this paper, firstly, we explicitly consider server available times in our scheduling model, which brings the work closer to reality. Secondly, we design a multi-installment scheduling strategy for this scheduling problem at hand.

The remainder of this paper is organized as follows. Section 2 firstly gives a mathematical description of the divisible-load scheduling problem on networked systems with arbitrary sever available times, followed by the proposed multi-installment scheduling model. With this model, we accordingly design a heuristic algorithm in Section 3 to obtain an optimal load-partitioning scheme, which will be evaluated through experiments in Section 4. In the last section, the conclusions are shared.

## 2. Multi-Installment Scheduling Model with Server Available Times

**Figure 1:**
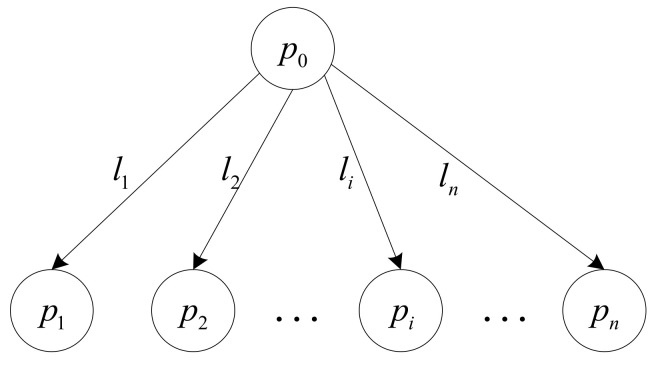
Heterogenous single-level tree network topology.

Let us consider n+1 servers connected in a single-level tree topology, as illustrated in Figure 1, where $p_{0}$ represents the master and $\left\{p_{1},p_{2},\ldots ,p_{n}\right\}$ denotes slave servers. The master $p_{0}$ is different from the master of the entire distributed system, but represents a server that stores the workload waiting to be processed and it does not participate in the workload computation but is only responsible for splitting the workload and distributing load partitions to slave servers in multiple installments, while slave servers are responsible for workload computation. The master is connected to each slave server through communication links, denoted as $\left\{l_{1},l_{2},\ldots ,l_{n}\right\}$. When all servers have completed their assigned load partitions, the entire workload is considered to have been completed. The time required from the first server accepting its assignment to the last server finishing load computation is called the makespan of the entire workload. Evidently, the shorter the makespan is, the better. That is, the goal of optimization is to pursue the shortest makespan for the entire workload. For ease of reading, the most commonly used notations in this paper are listed in Table 1.

The entire workload W to be processed arrives at the master at time t=0. First of all, before load transmission, it is necessary to establish a communication link between the master and each server, and experience a series of communication connections such as three handshakes. Due to the fact this period of time is relatively fixed, it can be regarded as a constant, which is called communication startup overhead, denoted as $e_{i}$ for link $l_{i}$. Let $g_{i}$ be the time required for link $l_{i}$ to transmit unit size of workload. Hence, it takes time $e_{i}+xg_{i}$ for link $l_{i}$ to transmit a workload with size x. After receiving load partitions from the master, each server needs to start specific components or programs needed for load computation. This startup time can be regarded as a constant, called the computation startup overhead, denoted as $f_{i}$ for server $p_{i}$. Let $w_{i}$ be the time required for server $p_{i}$ to finish computing the unit size of the workload. Hence, it takes time $f_{i}+xw_{i}$ for server $p_{i}$ to complete a workload with size x. The communication mode considered in this paper is a non-blocking mode. Hence, each server starts computing the moment it receives its assignment.

**Figure 2:**
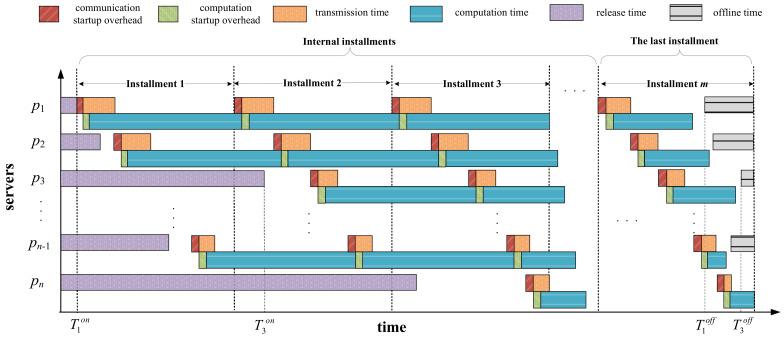
Multi-installment scheduling with server available times.

Figure 2 shows one possible Gantt chart for task-scheduling process on networked systems with arbitrary server available times. As can be seen, each server has different release and offline times, denoted as $T_{i}^{on}$ and $T_{i}^{off}$, respectively. The scheduling process consists of m installments, in which the first m−1 installments are denoted as internal installments. The last installment differs from the others since it ensures that all servers complete workload computation before their offline times and finish computing simultaneously as much as possible. If we do not consider server available times, to achieve the goal of minimizing the makespan, all servers are required to finish load computation at the same time. Otherwise, we can redistribute the partial load assigned to the server that finishes computing later to the server that completes first, thus reducing the makespan of the entire workload. This is referred to as the optimality principle in the DLT [14]. However, under the constraint that servers have heterogeneous offline times, the optimality principle is no longer applicable. In the last installment, the redundant part of the load assigned on the servers that do not meet the offline time constraint should be rescheduled to other servers that meet the time constraint. Moreover, for those servers that meet the offline time constraint, their load completion times should be as identical as possible to minimize the makespan.

The total size of the workload that all servers are expected to complete in each installment is V=W/m. The master assigns to server $P_{i}$ the loads $\alpha_{i}$ and $\beta_{i}$ in every internal installment and the last installment. The master transmits load partitions to one server at a time, that is, each server starts to receive its load partition after the master has finished sending divisions to its previous server. It is worth noting that each server must wait until its online time arrives to start receiving and computing its assigned load partitions, that is, the start time of each server is equal to the greater part of its online time and the time its previous server finished receiving its assignment. Similarly, each server must complete load computation in the last installment before its offline time arrives. Therefore, the makespan of the entire workload depends on the server that completes load computation the latest.

The goal of this paper is to find an optimal, if not a sub-optimal, load partitioning scheme that minimizes the makespan of the entire workload under the constraints of server available times.

### 2.1. Internal Installments

In order to ensure that each server has no time interval between any two adjacent internal installments, all servers must take the exact same time to process their assigned load fractions. Thus, we have

(1) $$f_{1}+\alpha_{1}w_{1}=f_{2}+\alpha_{2}w_{2}=\cdots =f_{n}+\alpha_{n}w_{n}$$

By Equation (1), $\alpha_{i}$ can be expressed by $\alpha_{1}$ as

(2) $$\alpha_{i}=\frac{\alpha_{1}w_{1}+f_{1}-f_{i}}{w_{i}}=\frac{w_{1}}{w_{i}}\alpha_{1}\;+\;\frac{f_{1}-f_{i}}{w_{i}},\;i=2,\;\ldots ,\;n.$$

Let us denote

(3) $$\Delta_{i}=\frac{w_{1}}{w_{i}}\;\mathrm{and}\;\Phi_{i}=\frac{f_{1}-f_{i}}{w_{i}}.$$

Substituting Equation (3) into (2) yields

(4) $$\alpha_{i}=\Delta_{i}\alpha_{1}+\Phi_{i},\;i=1,\;2,\;\ldots ,\;n.$$

Substituting Equation (4) into $\sum_{i=1}^{n}\alpha_{i}=V$, we have

(5) $$\alpha_{1}=\frac{V-\sum_{i=2}^{n}\Phi_{i}}{1+\sum_{i=2}^{n}\Delta_{i}}.$$

Let $\Phi_{1}=0$ and $\Delta_{1}=1$, we can simplify Equation (5) as

(6) $$\alpha_{1}=\frac{V-\sum_{i=1}^{n}\Phi_{i}}{\sum_{i=1}^{n}\Delta_{i}}.$$

Then, we obtain an optimal load partition for the internal installments as follows:

(7) $$\left\{\begin{array}{ll} \alpha_{1}=\frac{V-\sum_{i=1}^{n}\Phi_{i}}{\sum_{i=1}^{n}\Delta_{i}}, & \\ \alpha_{i}=\Delta_{i}\alpha_{1}+\Phi_{i}, & i=1,2,\ldots ,n. \end{array}\right.$$

Although the entire workload arrives at the master at time t=0, the scheduling process starts when the first server $p_{1}$ releases from its previous load computation at time $T_{1}^{on}$. If we neglect the online time constraint, the theoretical start time $T_{i}^{start}$ of server $p_{i}$ is as follows:

(8) $$\left\{\begin{array}{ll} T_{i}^{start}=T_{1}^{on}, & \\ T_{i}^{start}=T_{1}^{start}+\sum_{j=1}^{i-1}(e_{j}+\alpha_{j}g_{j}), & i=2,\ldots ,n. \end{array}\right.$$

Due to the online time constraint, servers may not be able to participate through the whole scheduling process. Hence, we need to identify which number of installments $u_{i}$ server $p_{i}$ can participate in the earliest as long as it meets the online time constraint.

(9) $$\left\{\begin{array}{ll} u_{1}=1, & \\ u_{i}=\left\lceil \frac{T_{i}^{on}-T_{i}^{start}}{f_{i}+\alpha_{i}w_{i}}\right\rceil , & i=2,\ldots ,n. \end{array}\right.$$

In theory, the total amount of workload that should be completed by all internal installments is (m−1)V. However, if there exists $u_{i}\neq 1$ with i=2,…,n, then server $p_{i}$ will miss several internal installments, resulting in the amount of workload (m−1)V not being completed entirely. The unfinished part of workload should be scheduled and completed in the last installment.

### 2.2. The Last Installment

The size of the workload waiting to be processed in the last installment can be written as

(10) $$V_{last}=V+\sum_{i=2}^{n}\left[(u_{i}-1)\alpha_{i}\right].$$

Here, we shall first derive an optimal load partition for the last installment without considering the offline time constraint. According to the optimal principle in the DLT, all servers should finish computing at the same time, so we have

(11) $$f_{i}+\beta_{i}w_{i}=\beta_{i}g_{i}+e_{i+1}+f_{i+1}+\beta_{i+1}w_{i+1},\;i=1,2,\ldots ,n-1.$$

From Equation (11), $\beta_{i+1}$ can be expressed by $\beta_{i}$

(12) $$\beta_{i+1}=\frac{w_{i}-g_{i}}{w_{i+1}}\beta_{i}+\frac{f_{i}-f_{i+1}-e_{i+1}}{w_{i+1}},\;\;i=2,\;\ldots ,\;n.$$

Let us define two new variables below for convenience.

(13) $$\lambda_{i+1}=\frac{w_{i}-g_{i}}{w_{i+1}}\;\mathrm{and}\;\theta_{i+1}=\frac{f_{i}-f_{i+1}-e_{i+1}}{w_{i+1}},\;i=1,\;\ldots ,\;n.$$

Simplifying Equation (12) by Equation (13), we have

(14) $$\beta_{i+1}=\lambda_{i+1}\beta_{i}+\theta_{i+1},\;i=1,\;\ldots ,\;n-1.$$

By recursively calculating Equation (14) by the iterative method, one can obtain the expression of $\beta_{i+1}$ by $\beta_{1}$

(15) $$\beta_{i+1}=\Psi_{i}\beta_{1}+\Omega_{\;i},\;i=1,\;2,\;\ldots ,\;n,$$

where

(16) $$\Psi_{i}=\prod_{j=2}^{i}\lambda_{j}\;\mathrm{and}\;\Omega_{\;i}=\sum_{j=2}^{i}\left(\theta_{j}\prod_{k=j+1}^{i}\lambda_{j}\right),\;\;i=1,\;2,\;\ldots ,\;n.$$

From the previous analysis, we know that the total amount of workload that all servers need to complete in the last scheduling is $V_{last}$, that is,

(17) $$\sum_{i=1}^{n}\beta_{i}=V_{last}.$$

Substituting Equation (15) into Equation (17), one can obtain an optimal load partition for the last installment without taking the offline time constraint into consideration.

(18) $$\left\{\begin{array}{ll} \beta_{1}=\frac{V_{last}-\sum_{i=2}^{n}\Omega_{\;i}}{1+\sum_{i=2}^{n}\Psi_{i}}, & \\ \beta_{i}=\Psi_{i}\beta_{1}+\Omega_{\;i}, & i=2,\ldots ,n. \end{array}\right.$$

Likewise, let $\Psi_{1}=1$ and $\Omega_{\;1}=0$. Then, Equation (18) can be simplified as

(19) $$\left\{\begin{array}{ll} \beta_{1}=\frac{V_{last}-\sum_{i=1}^{n}\Omega_{\;i}}{\sum_{i=1}^{n}\Psi_{i}}, & \\ \beta_{i}=\Psi_{i}\beta_{1}+\Omega_{\;i}, & i=1,\ldots ,n. \end{array}\right.$$

So far, without considering the offline time constraint, we have derived an optimal load partition for the last installment. However, since servers have heterogeneous offline times, it is necessary to adjust the value of $\beta_{i}$, so that the makespan of the entire workload could be the shortest under available time constraints.

### 2.3. Scheduling Model

Based on the actual load partitions for the last installment, the completion time $T_{i}^{end}$ of server $p_{i}$ can be written as follows:

(20) $$\left\{\begin{array}{ll} T_{i}^{end}=T_{1}^{on}+e_{1}+(m-1)(f_{1}+\alpha_{1}w_{1})+f_{1}+\beta_{1}w_{1}, & \\ T_{i}^{end}=T_{1}^{end}+\sum_{j=1}^{i-1}\left(e_{j}+\beta_{j}g_{j}\right)+e_{i}+f_{i}+\beta_{i}w_{i}, & i=2,\ldots ,n \end{array}\right.$$

Therefore, the makespan the entire workload is

(21) $$T(A,B,U)=\min\left\{\underset{1\leq i\leq n}{\max}\left\{T_{i}^{end}\right\}\right\}$$

where $A=(\alpha_{1},\alpha_{2},\ldots ,\alpha_{n})$, $B=(\beta_{1},\beta_{2},\ldots ,\beta_{n})$ and $U=(u_{1},u_{2},\ldots ,u_{n})$.

Here, we establish a new scheduling model aiming at a minimum makespan *T* under the available time constraints on networked systems.

(22) $$\underset{B}{\min}T(A,B,U)=\underset{B}{\min}\left\{\underset{1\leq i\leq n}{\max}\left\{T_{i}^{end}\right\}\right\}$$

subject to

(a) $0\leq \beta_{i}<V_{last},\;\sum_{i=1}^{n}\beta_{i}=V_{last},\;i=1,\;2,\;\ldots ,n.$
(b) $T_{i}^{end}\leq T_{i}^{off},\;i=1,\;2,\;\ldots ,n.$

where

(1) $\left\{\begin{array}{ll} T_{1}^{end}=T_{1}^{on}+e_{1}+(m-1)(f_{1}+\alpha_{1}w_{1})+f_{1}+\beta_{1}w_{1}, & \\ T_{i}^{end}=T_{1}^{end}+\sum_{j=1}^{i-1}\left(e_{j}+\beta_{j}g_{j}\right)+e_{i}+f_{i}+\beta_{i}w_{i}, & i=2,\ldots ,n. \end{array}\right.$
(2) $\left\{\begin{array}{ll} \alpha_{1}=\frac{V-\sum_{i=1}^{n}\Phi_{i}}{\sum_{i=1}^{n}\Delta_{i}}, & \\ \alpha_{i}=\Delta_{i}\alpha_{1}+\Phi_{i}, & i=1,2,\ldots ,n. \end{array}\right.$
(3) $V=\frac{W}{m},\;\Delta_{i}=\frac{w_{1}}{w_{i}},\;\Phi_{i}=\frac{e_{1}+f_{1}-e_{i}-f_{i}}{w_{i}},V_{last}=V+\sum_{i=2}^{n}\left[(u_{i}-1)\alpha_{i}\right].$
(4) $\left\{\begin{array}{ll} u_{1}=1, & \\ u_{i}=\left\lceil \frac{T_{i}^{on}-T_{i}^{start}}{f_{i}+\alpha_{i}w_{i}}\right\rceil , & i=2,\ldots ,n. \end{array}\right.$
(5) $\left\{\begin{array}{ll} T_{1}^{start}=T_{1}^{on}, & \\ T_{i}^{start}=T_{1}^{start}+\sum_{j=1}^{i-1}(e_{j}+\alpha_{j}g_{j}), & i=2,\ldots ,n. \end{array}\right.$

The optimization goal of the above model is to minimize the makespan of the entire workload. Although makespan T is a function of $A=(\alpha_{1},\alpha_{2},\ldots ,\alpha_{n})$, $B=(\beta_{1},\beta_{2},\ldots ,\beta_{n})$, and $U=(u_{1},u_{2},\ldots ,u_{n})$ according to Equation (22), we can obtain an optimal solution to A and U in Section 2.1, so only one set of variables, that is B, is involved in the proposed model. Moreover, this model contains two constraints. Constraint (a) means that every load partition assigned to severs in the last installment cannot be negative and that the total amount of workload completed by all servers in the last installment must be equal to $V_{last}$. Constraint (b) indicates that all servers must meet their corresponding offline time constraint. The reason for which the online time constraint is not reflected in the proposed model is that in Section 2.1 we have already taken online times into full consideration when obtaining an optimal to A and U.

## 3. Heuristic Scheduling Algorithm

In this section, a heuristic algorithm is designed to solve our proposed optimization model. It includes two strategies: a repair strategy and a local search strategy. The repair strategy is responsible for ensuring that all servers finish their load computation before their offline times, while the local search strategy ensures that servers can finish computing at the same time as much as possible (on the premise of satisfying available time constraints), so as to minimize the makespan of the entire workload.

### 3.1. Repair Strategy

The idea of the repair strategy is searching from $p_{1}$ to $p_{n}$ in turn to find the server that does not meet time constraints. If the completion time $T_{i}^{end}$ of server $p_{i}$ is greater than its offline time $T_{i}^{off}$, this means that the master has allocated too great an amount of assignment to this server. The extra amount of load will be assigned to the next server, that is, $p_{i+1}$. In this way, we only need to verify all servers once from beginning to end, and the time constraints of the proposed model will be satisfied after one round of repairment. In the worst case, all servers need to be fixed once.

Let us suppose there exists server $p_{i}$ that $T_{i}^{end}>T_{i}^{off}$. Let $\Delta K_{i}$ be the amount of load that needs to be assigned to server $p_{i+1}$. We have

(23) $$\Delta K_{i}=\frac{T_{i}^{end}-T_{i}^{off}}{w_{i}}.$$

We need to reschedule $\Delta K_{i}$ from server $p_{i}$ to server $p_{i+1}$. Let $\beta_{i}^{*}$ and $\beta_{i+1}^{*}$, respectively, be the updated amounts of load that is scheduled on servers $p_{i}$ and $p_{i+1}$ after repairing.

(24) $$\beta_{i}^{*}=\beta_{i}-\Delta K_{i}\;\mathrm{and}\;\beta_{i+1}^{*}=\beta_{i+1}+\Delta K_{i}.$$

Since load partitions assigned on servers $p_{i}$ and $p_{i+1}$ have been adjusted, their completion times are altered correspondingly, as follows.

(25) $$T_{i}^{end*}=T_{i}^{off}\;\mathrm{and}\;T_{i+1}^{end*}=T_{i+1}^{end}+(w_{i+1}-g_{i})\Delta K_{i}.$$

It is worth noting that since the master allocates less amount of load to server $p_{i}$ in the last installment, its transmission time becomes shorter. Conversely, as server $p_{i+1}$ has to undertake more load, its transmission takes a longer time. Due to the heterogeneity of the networked systems, the reduced transmission time of server $p_{i}$ is not necessarily equal to the increased transmission time of server $p_{i+1}$. This will cause the completion time $T_{j}^{end}$ of server $p_{j}$ with i+1<j≤n that schedules after $p_{i+1}$ changes as follows.

(26) $$T_{j}^{end*}=T_{j}^{end}+(g_{i+1}-g_{i})\Delta K_{i},\;i+1<j\leq n.$$

So far, the repair operation of server $p_{i}$ is completed. The repairment will continue to the next server $p_{i+1}$. If $p_{i+1}$ cannot complete its assignment before it reaches its offline time, we need to reschedule its overloaded partition to server $p_{i+2}$ and recalculate the completion time of all the servers behind $p_{i+2}$. Repeat this process until all servers have gone through the repair operation once.

**Figure 3:**
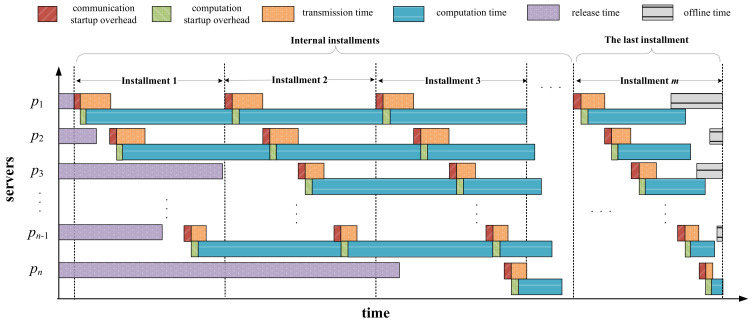
Multi-installment scheduling before applying repair strategy.

**Figure 4:**
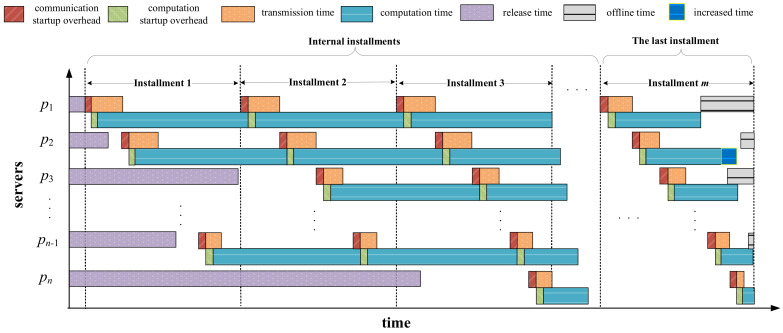
Multi-installment scheduling after applying repair strategy on the first server.

Figure 3 shows a case of multi-installment scheduling before applying the repair strategy. As can be seen from this figure, in the last installment, the completion time of server $p_{1}$ has exceeded its offline time. Hence, we have to reschedule the extra part of the load assigned on $p_{1}$ to server $p_{2}$, as illustrated in Figure 4. After being repaired, server $p_{1}$ finishes computing when its offline time arrives. Additionally, one can observe from Figure 4 that although server $p_{2}$ has been added with more load to process, it does not violate its offline time constraint. Therefore, we do not need to apply the repair operation on server $p_{2}$.

### 3.2. Local Search Strategy

After applying the repair strategy, all servers meet their offline time constraints, but the load partitioning scheme in the last installment at this time may not be an optimal solution to our proposed model. In this section, we shall put forward a local search strategy to enable as many servers as possible to finish computing at the same time while satisfying time constraints, thus minimizing the makespan of the entire workload.

The Idea of the local search strategy is to find the servers that finish computing the earliest and the latest, that is, to find the two servers with the largest gap in their completion times, and then balance the amount of load assigned on them to reduce the makespan. It is worth noting that the completion time of the earliest server must not have reached its offline time, because we need to allocate more load on it without violating its time constraint.

Let $p_{early}$ be the server that finishes computing the earliest and $p_{later}$ be the latest. After local search, their competition times turn into the same, that is $T_{early}^{end*}=T_{later}^{end*}$. Let us suppose that the amount of load that needs to be rescheduled from $p_{later}$ to $p_{early}$ is ΔV. We shall discuss the value of ΔV in two scenarios.

The first case is when early<later, which means that in the distribution sequence of the servers, server $p_{early}$ is in front of server $p_{later}$. Then, the following equations hold.

(27) $$\left\{\begin{array}{c} T_{early}^{end*}-T_{early}^{end}=w_{early}\Delta V,\;\;\;\;\\ T_{later}^{end}-T_{later}^{end*}=w_{later}\Delta V-g_{early}\Delta V,\\ T_{early}^{end*}=T_{later}^{end*}. \end{array}\right.$$

From Equation (27), one can derive the value of ΔV as

(28) $$\Delta V=\frac{T_{later}^{end}-T_{early}^{end}}{w_{early}+w_{later}-g_{early}}.$$

To avoid violating the offline time constraint, ΔV should be confined as follows.

(29) $$\Delta V=\min\left(\Delta V,\;\frac{T_{early}^{off}-T_{early}^{end}}{w_{early}}\right).$$

It is worth noting that since we allocate more load on server $p_{early}$, it needs more communication time to complete load transmission, which will definitely affect all subsequent servers behind $p_{early}$ in the distribution sequence and will increase their completion times. Similarly, we assign less load on server $p_{later}$, so its communication time becomes shorter, which will reduce the completion times of all subsequent servers behind $p_{later}$. Therefore, those servers lying between $p_{early}$ and $p_{later}$ in the distribution sequence are only affected by $p_{early}$. Their completion times will be changed into the following values.

(30) $$T_{i}^{end*}=T_{i}^{end}+g_{early}\Delta V,\;early<i<later.$$

The servers behind $p_{later}$ in the distribution sequence are affected by both $p_{early}$ and $p_{later}$. Their completion times will be altered as follows.

(31) $$T_{i}^{end*}=T_{i}^{end}+\left(g_{early}-g_{later}\right)\Delta V,\;later<i\leq n.$$

Observing Equations (30) and (31), we can come to the conclusion that all servers behind $p_{early}$ are affected. After local search, there may exist servers that violate time constraints, and thus we still need to apply the repair strategy on them.

**Figure 5:**
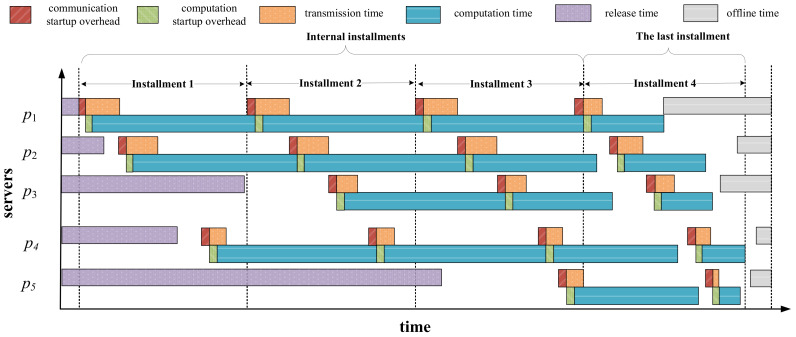
An example of the case when early<later.

Figure 5 shows an example of the case when early<later. As can be seen from this figure, server $p_{1}$ finishes computing at its offline time. Although it has the shortest completion time, server $p_{1}$ is not involved in the local search operation since it cannot be assigned more load. Except for $p_{1}$, server $p_{2}$ finishes computing the earliest while server $p_{4}$ finishes the latest. The load partitions allocated on them should be adjusted according to Equation (29). After load adjustment, the completion time of server $p_{3}$ will be enlarged, which can be updated by Equation (30). Meanwhile, the completion time of server $p_{5}$ will be affected by both servers $p_{2}$ and $p_{4}$, which can be calculated by Equation (31). Servers $p_{3}$ and $p_{5}$ may violate the offline time constraint, so they need to be verified or even repaired by the repair strategy.

The other case is when early>later, which means that server $p_{early}$ is behind server $p_{later}$ in the distribution sequence. Then, the following equations hold.

(32) $$\left\{\begin{array}{c} T_{early}^{end*}-T_{early}^{end}=w_{early}\Delta V-g_{later}\Delta V,\\ \begin{array}{l} T_{later}^{end}-T_{later}^{end*}=w_{later}\Delta V,\;\;\;\;\\ T_{early}^{end*}=T_{later}^{end*}.\;\;\;\; \end{array} \end{array}\right.$$

From Equation (32), one can derive the value of ΔV as follows.

(33) $$\Delta V=\frac{T_{later}^{end}-T_{early}^{end}}{w_{early}+w_{later}-g_{later}}.$$

Similarly, to avoid violating the offline time constraint, ΔV should be confined according to Equation (29). It is worth noting that since we allocate more load on server $p_{early}$, it needs more communication time to complete load transmission, which will definitely affect all subsequent servers behind $p_{early}$ in the distribution sequence and will increase their completion times. Similarly, we assign less load on server $p_{later}$, so its communication time becomes shorter, which will reduce the completion times of all subsequent servers behind $p_{later}$.

Those servers lying between $p_{later}$ and $p_{early}$ in the distribution sequence are only affected by $p_{later}$. Their completion times will be changed into the following values.

(34) $$T_{i}^{end*}=T_{i}^{end}-g_{later}\Delta V,\;later<i<early.$$

Those servers behind $p_{early}$ in the distribution sequence are affected by both $p_{early}$ and $p_{later}$. Their completion times will be altered as follows.

(35) $$T_{i}^{end*}=T_{i}^{end}+g_{early}\Delta V-g_{later}\Delta V,\;early<i\leq n.$$

Different from the first case, when early>later, the completion times of the servers between $p_{later}$ and $p_{early}$ are reduced after local search, as can be observed from Equation (34). Hence, they keep obeying the offline time constraint and need not to be repaired. By contrast, according to Equation (35), those servers behind $p_{early}$ in the distribution sequence may violate time constraints, and thus they need to be verified or even repaired.

Let $\beta_{early}^{*}$ and $\beta_{later}^{*}$, respectively, be the updated amounts of load rescheduled on servers $p_{early}$ and $p_{later}$ after local search. Then, we have

(36) $$\beta_{early}^{*}=\beta_{early}+\Delta V\;\mathrm{and}\;\beta_{later}^{*}=\beta_{later}-\Delta V.$$

**Figure 6:**
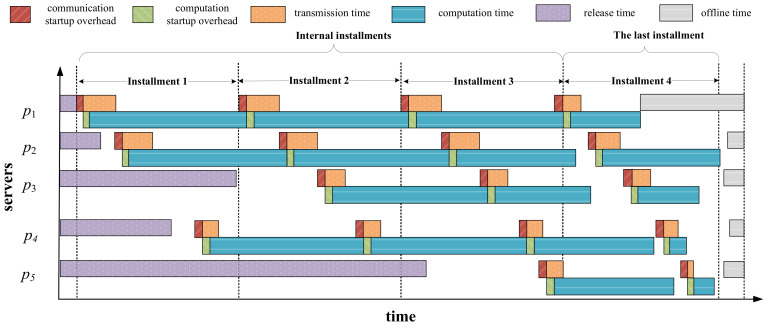
An example of the case when early>later.

Figure 6 shows an example of the case when early>later. Similar to Figure 5, except for server $p_{1}$, which is not involved in the local search operation, server $p_{2}$ finishes computing the latest while server $p_{4}$ finishes the earliest. The load partitions allocated to them should be adjusted according to Equation (29). After load adjustment, the completion time of server $p_{3}$ will be reduced, which can be updated by Equation (34). Meanwhile, the completion time of server $p_{5}$ will be affected by both servers $p_{2}$ and $p_{4}$, which can be calculated by Equation (35). Different from the case illustrated in Figure 5, only server $p_{5}$ needs to be verified or even repaired by the repair strategy.

### 3.3. Heuristic Algorithm

The proposed model only involves one set of variables, that is, the load partitioning scheme $B=(\beta_{1},\beta_{2},\ldots ,\beta_{n})$ for the last installment. To solve this model, we shall put forward in this section a heuristic algorithm, whose framework is given in Algorithm 1.
**Algorithm 1. Heuristic Scheduling Algorithm****Input**: Workload size W, number m of installments, parameters of servers, including $o_{i},g_{i},w_{i},f_{i},on_{i},off_{i}$ with i=1,2,…,n.**Output**: An optimal load-partitioning scheme (A,B,U), where $A=(\alpha_{1},\alpha_{2},\ldots ,\alpha_{n})$, $B=(\beta_{1},\beta_{2},\ldots ,\beta_{n})$ and $U=(u_{1},u_{2},\ldots ,u_{n})$.**Step 1**: Obtain an optimal load partition A for internal installments by Equation (7). **Step 2**: According to Equation (9), obtain the value of U, indicating which number of installments each server starts to participate in the internal installments.**Step 3**: Use Equation (10) to calculate out the amount of load $V_{last}$ that servers need to undertake in the last installment.**Step 4**: Initialize load partition B for the last installment by Equation (19).**Step 5**: Apply repair strategy to update B according to Equation (24).**Step 6**: Repeat applying local search strategy to update B according to Equation (36) until the completion time of every server is as identical as possible on the premise of meeting its offline time constraint.

Algorithm 1 first finds an optimal solution to the internal installments through steps 1 and 2. With the solutions of $A=(\alpha_{1},\alpha_{2},\ldots ,\alpha_{n})$ and $U=(u_{1},u_{2},\ldots ,u_{n})$, Algorithm 1 initializes B via steps 3 and 4. In order to meet the time constraints of the proposed model, Algorithm 1 repairs the load partitioning scheme through step 5, and then finds an optimal solution to B through step 6. Algorithm 1 stops when the competition time gap between any two servers who finish computing before their offline times is lesser than a small threshold.

## 4. Experiments and Analysis

In this section, we shall compare the model and algorithm proposed in this paper (denoted as MIS) with the existing single-installment scheduling model and algorithm based on server available times [36] (abbreviated as SIS), to prove that the proposed model and algorithm can achieve a shorter makespan.

In the following experiments, the total number of servers is set to be 15, that is, n=15. Table 2 lists the parameters of the heterogenous networked system, where $e_{i}$ is the communication startup overhead of server $p_{i}$, $g_{i}$ is the communication time of link $l_{i}$ to transmit the unit size of workload, $f_{i}$ is the computation startup overhead of server $p_{i}$, $w_{i}$ is the computation time of server $p_{i}$ to finish computing unit size of workload, $T_{i}^{on}$ is the online time of server $p_{i}$ while $T_{i}^{off}$ is its offline time. These values listed in Table 2 are commonly used in the DLT field [10].

**Figure 7:**
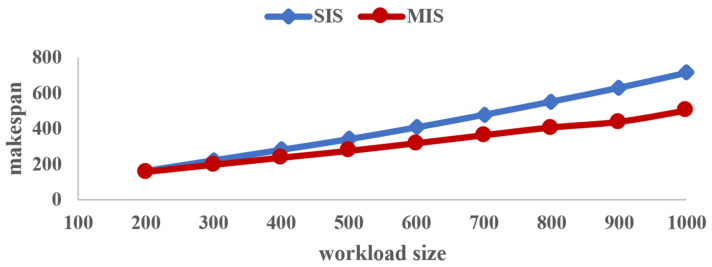
Makespan of the entire workload vs. workload size.

Table 3 gives the makespan obtained by the two algorithms SIS and MIS with different workloads. In order to visually demonstrate their difference, Figure 7 shows the curve of makespan vs. workload size.

As can be seen from Figure 7, first of all, no matter how great the workload is, the makespan obtained by the proposed algorithm MIS (represented in red) is always less than that obtained by the compared algorithm SIS (marked in blue). That is to say, our proposed heuristic algorithm can obtain a shorter makespan of workloads and improve the workload processing efficiency when addressing scheduling problems employing heterogeneous servers with arbitrary available times.

Secondly, one can observe from Figure 7 that with an increase in the workload size, the makespan obtained by either of the two algorithms SIS and MIS becomes larger and larger. Moreover, it is worth noting that the time gap between the two algorithms is greatly increased, which indicates that the proposed algorithm has superior performance in solving large-scale divisible-load scheduling problems in the era of big data.

## 5. Conclusions

The goal of this paper is to obtain a minimized makespan for a large-scale workload processed on networked systems with heterogeneous servers that have arbitrary available times. The goal was successfully achieved via three distinct efforts. First, we proposed a new multi-installment scheduling model with server availability constraints. The scheduling process was divided into two main parts: internal installments and the last installment, so as to simplify the scheduling process. Second, taking full advantage of server release times, we obtained an optimal load-partitioning scheme for the internal installments through strict mathematical derivation. Third, we put forward an effective heuristic algorithm to obtain an optimal load-partitioning scheme for the last installment. The proposed heuristic algorithm is effective owing to the two well-designed optimization strategies contained—a repair strategy and a local search strategy. The repair strategy ensures that all servers complete their load computation before offline times, thus satisfying the available time constraints. The local search strategy guarantees that servers can finish computing at the same time as much as possible on the premise of satisfying available time constraints, so as to minimize the makespan of the entire workload. Finally, the experimental results show that, compared with the existing scheduling algorithm, the proposed one is superior in obtaining a shorter makespan, especially when addressing large-scale scheduling problems in the era of big data. Extensions to this work can consider server outages together with available times, and this is expected to be an open-ended problem at this juncture.

## Figures and Tables

**Table 1:** Notations and their meanings.

Notations	Meanings
n	Total number of servers involved in workload computation
$\left\{p_{1},p_{2},\ldots ,p_{n}\right\}$	Servers involved in workload computation
$\left\{l_{1},l_{2},\ldots ,l_{n}\right\}$	Communication links connected the master and the servers
W	Total size of the workload to be processed on a networked system
$e_{i}$	Communication startup overhead of server $p_{i}$
$f_{i}$	Computation startup overhead of server $p_{i}$
$g_{i}$	Communication time of link $l_{i}$ to transmit unit size of workload
$w_{i}$	Computation time of server $p_{i}$ to finish computing unit size of workload
$T_{i}^{on}$	Online time of server $p_{i}$
$T_{i}^{off}$	Offline time of server $p_{i}$
$A=(\alpha_{1},\alpha_{2},\ldots ,\alpha_{n})$	$\alpha_{i}$ is the load partition assigned on server $p_{i}$ in each internal installment
$B=(\beta_{1},\beta_{2},\ldots ,\beta_{n})$	$\beta_{i}$ is the load partition assigned on server $p_{i}$ in the last installment
$U=(u_{1},u_{2},\ldots ,u_{n})$	$u_{i}$ indicates which number of installments server $p_{i}$ starts to participate in the internal installments
$T_{i}^{start}$	Theoretical start time of server $p_{i}$ when sever online time constraint is not considered
$T_{i}^{end}$	Completion time of server $p_{i}$
$T_{i}^{end*}$	Updated completion time of server $p_{i}$ after local search or repair strategy
T	Makespan of the entire workload

**Table 2:** Parameters of the heterogeneous networked system.

Servers	$e_{i}$	$g_{i}$	$f_{i}$	$w_{i}$	$T_{i}^{on}$	$T_{i}^{off}$
*p*_1_	2.51	0.97	0.52	5.19	25.17	1045.99
*p*_2_	2.04	0.51	0.24	3.49	36.94	341.67
*p*_3_	1.37	0.87	0.14	4.24	86.74	1124.78
*p*_4_	2.02	0.26	0.28	5.53	41.35	1273.56
*p*_5_	3.25	0.74	0.35	5.16	43.35	379.53
*p*_6_	4.29	0.93	0.19	4.58	34.34	957.27
*p*_7_	1.78	0.72	0.46	3.41	68.52	431.68
*p*_8_	3.64	0.24	0.16	6.75	32.92	862.64
*p*_9_	3.11	0.68	0.22	6.32	63.47	529.62
*p*_10_	3.25	0.61	0.39	4.91	45.67	1446.8
*p*_11_	4.29	0.42	0.26	3.89	23.46	623.24
*p*_12_	2.75	0.86	0.13	5.41	32.7	1682.13
*p*_13_	1.43	0.69	0.25	6.07	56.92	716.17
*p*_14_	1.78	0.29	0.43	4.89	56.42	1872.37
*p*_15_	5.91	0.36	0.32	3.28	36.52	798.47

**Table 3:** Experimental results—comparison between SIS and MIS.

Workload Size	SIS	MIS
200	164.03	156.902
300	223.141	196.93
400	282.223	236.554
500	341.755	274.961
600	408.189	318.141
700	478.732	362.966
800	553.166	406.991
900	630.684	438.42
1000	714.867	502.245
